# Unexpected Course of Reaction Between (1*E*,3*E*)-1,4-Dinitro-1,3-butadiene and N-Methyl Azomethine Ylide—A Comprehensive Experimental and Quantum-Chemical Study

**DOI:** 10.3390/molecules29215066

**Published:** 2024-10-26

**Authors:** Mikołaj Sadowski, Karolina Kula

**Affiliations:** Department of Organic Chemistry and Technology, Cracow University of Technology, Warszawska 24, 31-155 Cracow, Poland; mikolaj.sadowski@doktorant.pk.edu.pl

**Keywords:** nitrodiene, azomethine ylide, cycloaddition, synthesis, spectral characteristics, MEDT, ADME, PASS

## Abstract

In recent times, interest in the chemistry of conjugated nitrodienes is still significantly increasing. In particular, the application of these compounds as building blocks to obtain heterocycles is a popular object of research. Therefore, in continuation of our research devoted to the topic of conjugated nitrodienes, experimental and quantum-chemical studies of a cycloaddition reaction between (1*E*,3*E*)-1,4-dinitro-1,3-butadiene and N-methyl azomethine ylide have been investigated. The computational results present that the tested reaction is realized through a *pdr*-type polar mechanism. In turn, the experimental study shows that in a course of this cycloaddition, only one reaction product in the form of 1-methyl-3-(*trans*-2-nitrovinyl)-Δ^3^-pyrroline is created. The constitution of this compound has been confirmed via spectroscopic methods. Finally, ADME analysis indicated that the synthesized Δ^3^-pyrroline exhibits biological potential, and it is a good drug candidate according to *Lipinski*, *Veber* and *Egan* rules. Nevertheless, PASS simulation showed that the compound exhibits weak antimicrobial, inhibitory and antagonist properties. Preliminary in silico research shows that although the obtained Δ^3^-pyrroline is not a good candidate for a drug, the presence of a nitrovinyl moiety in its structure indicates that the compound is an initial basis for further modifications.

## 1. Introduction

The chemistry of unsaturated organic nitro compounds has developed significantly over the last twenty years [1]. One of the most important and popular examples of this group of organic compounds is conjugated nitroalkenes (CNAs) [2,3]. This is because of their unique properties. Firstly, CNAs can be prepared via a simple protocol based on the decomposition of saturated nitro compounds [4] or nitration of unsaturated molecular systems [5]. Moreover, these compounds exhibit many biological properties. The most important of them include antibacterial [6,7] and antifungal [8] activities.

CNAs are extremely useful and common in many reactions. This is due to the presence in the structures of these compounds an active nitro group that can be easily converted to other connections such as hydroxylamines, oximes, nitriles and many others [9]. CNAs are also readily used in *Michael* reactions to obtain of biologically active molecules [10,11]. However, the most popular application of CNAs is in synthesis of cyclic compounds. For this purpose, the protocol of the cycloaddition reaction is widely applied [12,13]. As a result, carbo- and heterocyclic compounds such as nitrofunctionalized derivatives of cyclopentane [14], cyclohexene [15,16], as well as pyrazolines [17,18], isoxazolines [19,20,21,22] and also pyrrolidine [23,24] and others are obtained.

**Figure 1:**
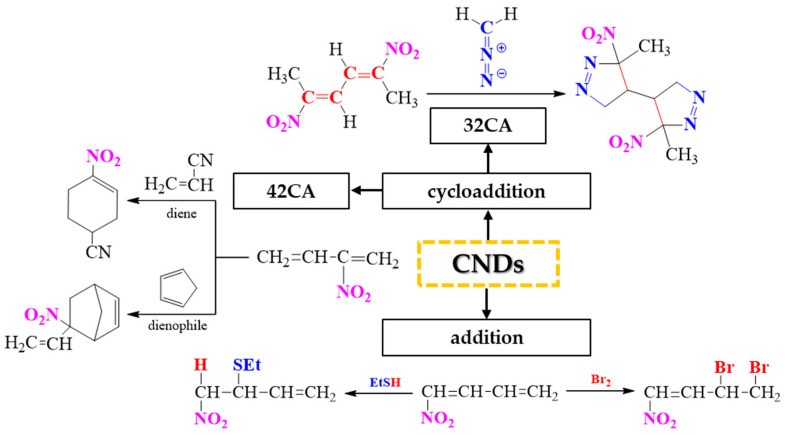
Examples of the application of CNDs in organic synthesis.

All in all, CNAs constitute one of the better-known and tested unsaturated nitro compounds, without a doubt. However, in recent times, another class of nitro unsaturated organic compounds, namely conjugated nitrodienes (CNDs), are gaining growing interest [25,26]. Due to their structure, these compounds offer a much greater possibility of *trans*formation compared to CNAs. First of all, CNDs can be successfully applied, via an addition reaction, in the synthesis of nitro derivatives of ethers, sulphides, as well as halogen compounds [25,26,27] (Figure 1). An application of CNDs that is still being intensively explored is their use in the synthesis of heterocycles. Due to their structure and presence in the structure of the conjugated system, CNDs are used in [4 + 2] cycloaddition (42CA) reactions. In these reactions, they usually play the role of a diene, but cases of their application as a dienophile are also known [28] (Figure 1). What is more, the [3 + 2] cycloaddition (32CA) of CNDs can lead to two interesting structural connections. The first one is a ring with a highly reactive nitrovinyl substituent [29]. However, 32CA can take place on both of carbon–carbon double bonds. As a consequence, bis-cyclic systems can be obtained (Figure 1) [28]. These types of compounds are desirable due to their potential use in electronics, in medicine [30], as well as in optoelectronics [31] as highly efficient photovoltaic materials [32] or solar cells [33].

Despite the attractiveness and wide range of possibilities in the synthesis of cyclic compounds provided by the CNDs, the chemistry of these compounds is still little known. Most of the available research focuses on the addition reactions to obtain ethers, sulphides and also halogen derivatives [25,26,27]. Therefore, the use of CNDs as building blocks to obtain heterocyclic systems is an attractive course of research and determines one of the most promising trends in current organic synthesis, in particular, taking into account the fact that some of these compounds have biological potential [34] and therefore can be successfully used as active substances in medicine [35] and agriculture [36]. 

Considering the above, in continuation of our research of CNDs and their application in synthesis of heterocycles [37], comprehensive experimental and computational studies of the 32CA reaction with the participation of (1*E*,3*E*)-1,4-dinitro-1,3-butadiene (**1**) have been performed. In the role of a three-atom component (TAC) [38], an N-methyl azomethine ylide (**2**) was used. Recently, this compound was investigated several times in 32CA reactions with CNAs [23,39].

**Scheme 1:**
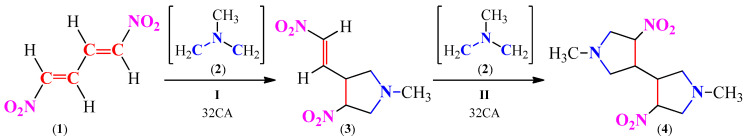
Theoretically possible reaction paths of 32CA between nitrodiene (**1**) and ylide (**2**).

Theoretically, the presented reaction can take place only on one carbon–carbon double bond of diene (product **3**), but in practice, this type of reaction is realized on both carbon–carbon double bonds of diene [40]. So, the more expected product is the bis-cyclic system (product **4**) (Scheme 1). 

The presented research was divided in four sections. First, an analysis of electronic properties of used reagents, namely nitrodiene (**1**) and ylide (**2**) based on Molecular Electron Density Theory (MEDT) [41], was carried out. Then, the titled reaction was tested synthetically. The obtained reaction mixture was separated. Next, the obtained product was isolated and purified. The constitution of the molecule was confirmed based on physicochemical and spectral characteristics. In the third part of the manuscript, the creation of the obtained product was analysed via quantum-chemical tools. Finally, in order to evaluate the biological potential of the obtained product in silico analyses—including physicochemical descriptors, pharmacokinetic properties, druglike nature and medicinal chemistry friendliness based on selected aspects of Absorption, Distribution, Metabolism and Excretion (ADME) [42] studies and Prediction of Activity Spectra for Substances (PASS) [43]—prediction was performed.

## 2. Results and Discussion 

### 2.1. Study of Electronic Properties of (1E,3E)-1,4-Dinitro-1,3-butadiene (***1***) and N-Methyl Azomethine Ylide (***2***) Based on MEDT

In order to understand the electronic nature of the tested reagents (**1**) and (**2**) as well as to better understand the kind of correlation between these compounds, computational studies were carried out. For this purpose, the selected aspects of MEDT [41] were applied.

#### 2.1.1. Study of the Electronic Properties for Reagents **1** and **2** Based on ELF, NPA and MEP

Firstly, a topological analysis of the Electron Localization Function (ELF) [44] together with a Natural Population Analysis (NPA) [45,46] and molecular electrostatic potential (MEP) [47] for (1*E*,3*E*)-1,4-dinitro-1,3-butadiene (**1**) and N-methyl azomethine ylide (**2**) were performed in order to characterize the electronic structures of the substances. The applied approach is commonly used in research of electron density distribution in molecules and allows us to characterize both the electronic structures of compounds as well as to predict their reactivity in chemical processes. 

**Figure 2:**
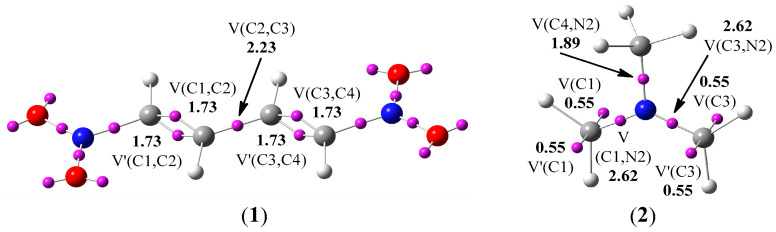
B3LYP/6-31G(d) ELF attractor positions of the core and valence basins for nitrodiene (**1**) and ylide (**2**) together with the most significant ELF valence basin populations. The ELF attractors are shown as pink spheres. The electron populations are given as the average number of electrons [e].

**Figure 3:**
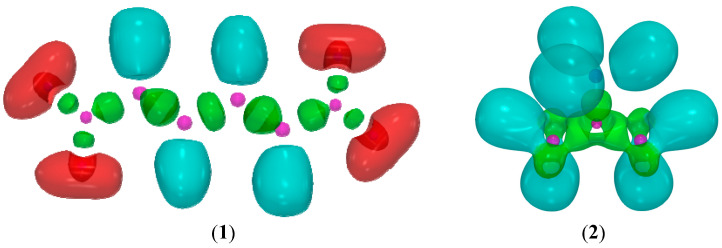
B3LYP/6-31G(d) ELF localization domains for nitrodiene (**1**) and ylide (**2**) represented at an isosurface value of ELF = 0.75. For ELF localization domains, protonated basins are shown in blue, monosynaptic basins in red, disynaptic basins in green and core basins in magenta.

The ELF attractor positions of the core and valence basins together with the most relevant valence basin populations for (1*E*,3*E*)-1,4-dinitro-1,3-butadiene (**1**) and N-methyl azomethine ylide (**2**) are shown in Figure 2, while the ELF localization domains of molecules (**1**) and (**2**) are given in Figure 3.

The ELF topological analysis of the most significant fragment for (1*E*,3*E*)-1,4-dinitro-1,3-butadiene (**1**) shows the presence of two complexes of two pairs of disynaptic basins (Figure 3), namely V(C1,C2) and V’(C1,C2), integrating a total electron population of 3.46 e and also V(C3,C4) and V’(C3,C4), integrating the same value of total electron population of 3.46 e (Figure 2). The presence of these disynaptic basins is associated with somewhat depopulated C1-C2 and C3-C4 double bonds. In turn, the presence of the disynaptic basin (Figure 3) V(C2,C3), integrating a population of 2.23 e (Figure 2), is associated with a slightly overpopulated C2-C3 single bond. All presented results confirm the standard structure of (1*E*,3*E*)-1,4-dinitro-1,3-butadiene (**1**). 

The ELF topological analysis of the most significant fragment for N-methyl azomethine ylide (**2**) shows the presence of two complexes of two pairs of monosynaptic basins (Figure 3), namely V(C1) and V’(C1), integrating a total electron population of 1.10 e, located at the carbon C1 atom of the TAC fragment, as well as V(C3) and V’(C3), integrating the same value of total electron population of 1.10 e, located at the C3 carbon atom of the TAC fragment (Figure 2). Other important ELF attractors are those of two disynaptic basins (Figure 3), namely V(C1,N2) and V(C3,N2), integrating an electron population of 2.62 e in each case (Figure 2), associated with a highly depopulated C1-N2 and C3-N2 one-and-a-half-order bonds, as well as a disynaptic basin (Figure 3), namely V(C4,N2), integrating an electron population of 1.89 e (Figure 2), associated with a slightly depopulated C4-N2 single bond. ELF topological analysis of N-methyl azomethine ylide (**2**) indicates that this TAC can be classified as an N-centred ally type of pseudodiradical electronic structure [48,49] participating in *pdr*-type 32CA reactions [50,51].

**Figure 4:**
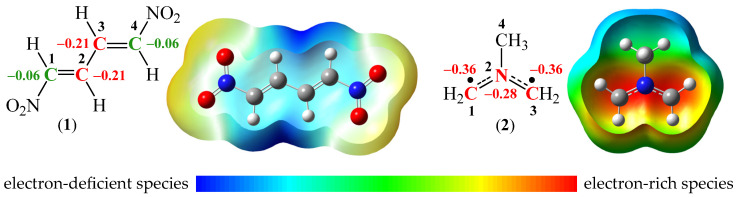
B3LYP/6-31G(d) proposed ELF-based Lewis-like structures with the natural atomic charges for nitrodiene (**1**) and ylide (**2**) as well as the molecular electrostatic potential maps. Negative charges are coloured in red, while negligible charges are coloured in green. Natural atomic charges are given as the average number of electrons [e].

Based on ELF analysis, we propose Lewis-like structures. For this purpose, NPA and MEP studies were carried out. The proposed structures together with the natural atomic charges as well as the molecular electrostatic potential maps for (1*E*,3*E*)-1,4-dinitro-1,3-butadiene (**1**) and N-methyl azomethine ylide (**2**) are shown in Figure 4.

The NPA analysis of (1*E*,3*E*)-1,4-dinitro-1,3-butadiene (**1**) indicates a moderate negative distribution located at the central C2 and C3 carbon atoms, which form a single bond. For molecule (**1**), the natural atomic charges for both C2 and C3 carbon atoms are −0.21 e (Figure 4). In turn, a different situation is observed in the case of terminal C1 and C4 carbon atoms, directly connected with nitro groups. In this case the natural atomic charges for both C1 and C4 carbon atoms are strongly depleted of electrons to a value typical of neutral regions, namely −0.06 e (Figure 4).

In turn, the NPA analysis of N-methyl azomethine ylide (**2**) indicates a strongly negative distribution located along pseudodiradical moiety. For molecule (**2**), simultaneously, both the C1 and C3 carbon atoms are represented by highly negative regions, namely −0.36 e (Figure 4). What is more, in molecule **2,** for an N2 nitrogen atom, a slight decrease in electron-rich species is noticeable, with a natural atomic charge of −0.28 e (Figure 4).

The presented results correlate well with MEP. In particular, the analysis of the surface for nitrodiene (**1**) shows a slightly negative electrostatic potential around the carbon atoms (in light blue), which increases to a moderately electronegative region within the vicinity of the hydrogen atoms (in light green) (Figure 4). In turn, in the ylide (**2**), there is a highly negative electrostatic potential around the pseudodiradical centre (in red) and a poorly electropositive region within the methyl moiety (from green to blue) (Figure 4).

#### 2.1.2. Analysis of Reactivity Indices for Reagents **1** and **2** According to CDFT

In the next part of the computational study, an analysis of reactivity indices for (1*E*,3*E*)-1,4-dinitro-1,3-butadiene (**1**) and N-methyl azomethine ylide (**2**) were carried out. For this purpose, the protocol of Conceptual Density Functional Theory (CDFT) was applied. In general, the CDFT is an important approach in understanding the reactivity of molecules, widely used in modern chemistry. The theory is to connect well-established chemical concepts, like electronic chemical potential μ, chemical hardness η and chemical softness S, with the electronic structure of a molecule. Based on these, the indication of global electronic properties of substrates, such as global electrophilicity ω and global nucleophilicity N for molecules, can be established [52]. As an effect, it is possible to assign addends a role of either an electrophile or a nucleophile in studied reactions [53,54,55]. Furthermore, with the application of Parr functions, not only global but also local electronic properties of a molecule can be estimated, thus allowing us to predict reactivity of molecules in the studied reactions, based only on the substrates’ structures [56,57]. 

**Figure 5:**
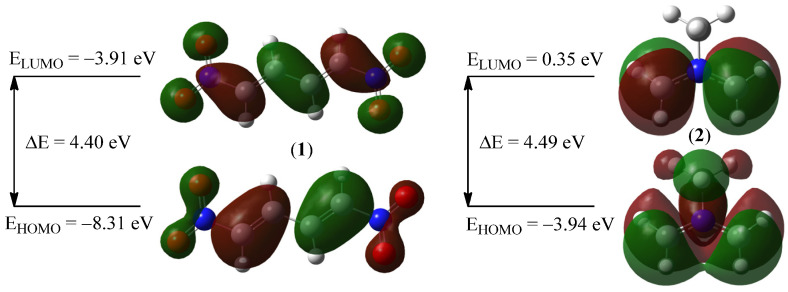
B3LYP/6-31G(d) HOMO–LUMO energy gap ΔE diagram for nitrodiene (**1**) and ylide (**2**).

**Figure 6:**
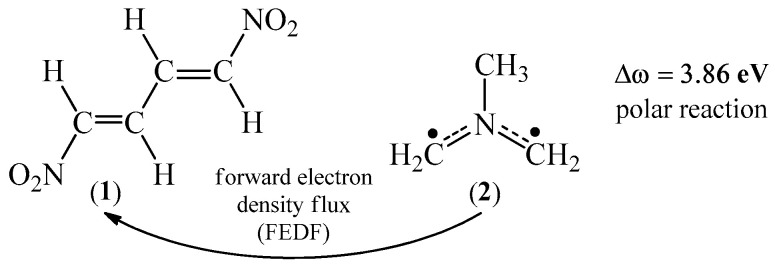
B3LYP/6-31G(d) visualization of the flux of the electron density between nitrodiene (**1**) and ylide (**2**) together with the driving force of the process Δω parameter.

**Figure 7:**
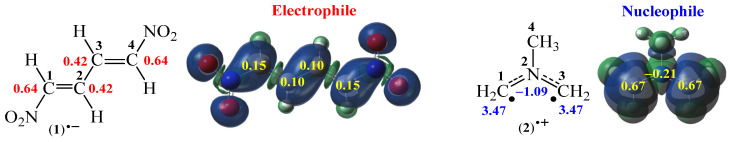
B3LYP/6-31G(d) local electronic properties for nitrodiene (**1**) and ylide (**2**) presented as three-dimensional (3D) representations of Mulliken atomic spin densities for radical anion of **1**^−^ and radical cation **2**^+^, together with the electrophilic P_k_^+^ and the nucleophilic P_k_^−^ Parr functions values (given in yellow), as well as the indices of the local electrophilicity ω_k_ of **1**^−^ (given in red, in eV) and the local nucleophilicity N_k_ of **2**^+^ (given in blue, in eV).

The global reactivity indices for (1*E*,3*E*)-1,4-dinitro-1,3-butadiene (**1**) and N-methyl azomethine ylide (**2**), together with information about HOMO and LUMO energy, are given in Table 1. The HOMO–LUMO energy gap diagrams of reagents (**1**) and (**2**) is presented in Figure 5, while their local electronic properties are shown in Figure 7. Additionally, the selected aspects of the considered reaction between nitrodiene (**1**) and ylide (**2**), such as the flux of the electron density as well as the polarity of the reaction, are included in Figure 6.

The computed HOMO energy of (1*E*,3*E*)-1,4-dinitro-1,3-butadiene (**1**) is −8.31 eV and the computed LUMO energy for this diene (**1**) is −3.91 eV (Table 1). The strongly negative energy values for these frontier molecular orbitals are caused by the presence of two nitro groups [37]. Consequently, the HOMO–LUMO energy gap (ΔE) is 4.40 eV (Figure 5). In the case of N-methyl azomethine ylide (**2**), values of both frontier molecular orbitals are strongly increased. In particular, the computed HOMO energy is −3.94 eV, and the computed LUMO energy for this ylide (**2**) is 0.35 eV (Table 1). Nevertheless, the HOMO–LUMO energy gap (ΔE) is practically identical, namely 4.49 eV (Figure 5). 

The HOMO–LUMO energy gap is an important stability index as it explains the charge transfer interactions within the molecule and is also useful in determining molecular electronic transport properties. A molecule with a high frontier molecular orbital HOMO–LUMO energy gap ΔE is characterized by low chemical reactivity and simultaneously high kinetic stability. The phenomenon is related to the high excitation energy between the high-lying LUMO and the low-lying HOMO energy levels [58]. The estimated energy gaps ΔE for (1*E*,3*E*)-1,4-dinitro-1,3-butadiene (**1**) and N-methyl azomethine ylide (**2**) mean that both reagents are characterized by similar stability and also similar reactivity in chemical reactions.

Table 1 shows that the electronic chemical potential μ of the (1*E*,3*E*)-1,4-dinitro-1,3-butadiene (**1**), μ = −6.11 eV, is significantly lower in compared to the N-methyl azomethine ylide (**2**), μ = −1.79 eV. This means that the flux of the electron density will take place from ylide (**2**) to nitrodiene (**1**) (Figure 6). Therefore, the considered reaction between reagents (**1**) and (**2**) should be classified as the forward electron density flux (FEDF) [59,60]. 

Polar reactions require the participation of good electrophiles as well as good nucleophiles [53]. Useful information about the polarity of a reaction may be obtained from the difference in the global electrophilicity ω indices between reagents involved in the reaction in a form of parameter which assesses driving force of the process Δω [61]. In general, the reactions can be considered as polar processes when Δω > 1 eV [62,63]. The computed global electrophilicity ω index of (1*E*,3*E*)-1,4-dinitro-1,3-butadiene (**1**) is 4.24 eV, and the computed global electrophilicity ω index of N-methyl azomethine ylide (**2**) is 0.38 eV (Table 1). Therefore, the Δω parameter of the reaction between reagents (**1**) and (**2**) is 3.86 eV (Figure 6). Based on this information, it should be concluded that the considered reaction can be classified as a polar process.

The computed global electrophilicity [64] ω index of (1*E*,3*E*)-1,4-dinitro-1,3-butadiene (**1**) is 4.24 eV, and the calculated global nucleophilicity [65] N index for this compound (**1**) is 0.81 eV (Table 1). These values lead us to conclude that nitrodiene (**1**) can be classified as a super strong electrophile and marginal nucleophile in a polar reaction, within the electrophilicity and nucleophilicity scale [53,66]. In turn, for N-methyl azomethine ylide (**2**), the computed global electrophilicity [64] ω index is 0.38 eV, and the calculated global nucleophilicity [65] N index for this compound (**2**) is 5.18 eV (Table 1). These values give the conclusion that ylide (**2**) can be classified as a marginal electrophile and super strong nucleophile in a polar reaction, within the electrophilicity and nucleophilicity scale [53,66]. Overall, in the considered reaction, it can be assumed that the (1*E*,3*E*)-1,4-dinitro-1,3-butadiene (**1**) will participate as an electrophilic component, while the N-methyl azomethine ylide (**2**) will play the role of a nucleophilic agent. 

The presented analysis leads to an important conclusion, because thanks to this information, it is possible to define the most electrophilic centre of the electrophile and the most nucleophilic centre of the nucleophile [56,57]. To characterize the most electrophilic centres for (1*E*,3*E*)-1,4-dinitro-1,3-butadiene (**1**) and the most nucleophilic centres for N-methyl azomethine ylide (**2**), the electrophilic P_k_^+^ and nucleophilic P_k_^−^ Parr functions together with local electrophilicity ω_k_ and local nucleophilicities N_k_ of reagents (**1**) and (**2**) were investigated (Figure 7).

The analysis of the electrophilic P_k_^+^ Parr function [57] of (1*E*,3*E*)-1,4-dinitro-1,3-butadiene (**1**) indicates that the terminal C1 and C4 carbon atoms are the most electrophilic centres of this species, both presenting the value P_C_^+^ = 0.15. In these atoms, the value of the local electrophilicity ω_k_ index is ω_C_ = 0.64 eV (Figure 7). In turn, electrophilic P_k_^+^ Parr functions for the centric C1 and C4 carbon atoms of this compound (**1**) are noticeably reduced to P_C_^+^ = 0.10 (ω_C_ = 0.42 eV) (Figure 7). This is related to the direct neighbourhood of strongly electron-withdrawing nitro groups, which cause a significant increase in local electrophilic properties in nitrodiene (**1**). On the other hand, the analysis of nucleophilic P_k_^−^ Parr functions [57] of N-methyl azomethine ylide (**2**) shows that both the C1 and C3 carbon atoms of ylide moiety are the most nucleophilic centres of this species, both presenting the value P_C_^−^ = 0.67. In these atoms, the value of the local nucleophilicity N_k_ index is N_C_ = 3.47 eV (Figure 7). In turn, the nucleophilic P_k_^−^ Parr function for another atom of the TAC moiety of this compound (**2**), which is the N2 nitrogen atom, is significantly reduced to P_N_^−^ = −0.21 (ω_N_ = −1.09 eV) (Figure 7). Therefore, this atom of the ylide (**2**) does not play a major role in interactions with nitrodiene (**1**). 

### 2.2. Synthetic Aspects of Reaction Between (1E,3E)-1,4-Dinitro-1,3-butadiene (***1***) and N-Methyl Azomethine Ylide (***2***)

#### 2.2.1. Protocol Synthesis Details of Necessary Reagents (**1**) and (**2**)

**Scheme 2:**
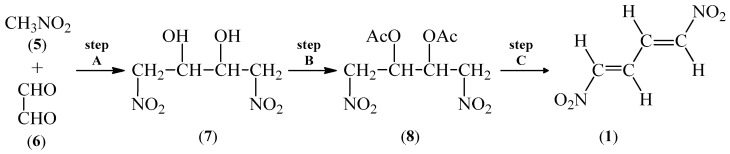
Method of the three-step synthesis of (1*E*,3*E*)-1,4-dinitro-1,3-butadiene (**1**).

At the beginning, a synthesis of the necessary reagents was carried out. The (1*E*,3*E*)-1,4-dinitro-1,3-butadiene (**1**) was obtained first. Generally, in the literature, two synthesis methods of this nitrodiene (**1**) are available [25]. In the presented study, a three-step synthesis protocol was used (Scheme 2), described in detail in our last work [37]. Firstly, the condensation reaction between nitromethane (**5**) and glyoxal (**6**) in alkaline solution was carried out (Step A). As a result, the 1,4-dinitrobutane-2,3-diol (**7**) was obtained. Next, the nitroalcohol (**7**) was converted to nitroester (**8**) via an esterification reaction through acetyl chloride in glacial acetic acid (Step B). Finally, the obtained 2,3-diacetoxy-1,4-dinitrobutane (**8**) was transformed via thermal dehydro-acetylation reaction using potassium bicarbonate in chloroform (Step C). As a result, the (1*E*,3*E*)-1,4-dinitro-1,3-butadiene (**1**) was obtained. 

**Scheme 3:**
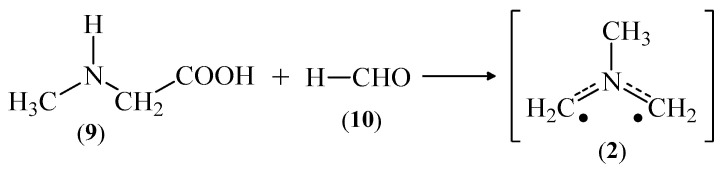
*In situ* synthesis method of N-methyl azomethine ylide (**2**).

In turn, based on examples of cycloaddition reactions involving N-methyl azomethine ylide (**2**) described in the literature [23,39], the synthesis of this compound (**2**) was performed directly in the reaction mixture, from commercially available sarcosine (**9**) and formaldehyde (**10**) (Scheme 3). 

#### 2.2.2. Protocol Details of Reaction Between Nitrodiene (**1**) and Ylide (**2**)

In the next part of the research, the synthetic study on the titled reaction between (1*E*,3*E*)-1,4-dinitro-1,3-butadiene (**1**) and N-methyl azomethine ylide (**2**) was carried out (Scheme 1). Based on information about similar protocols for the synthesis of heterocyclic compounds by cycloaddition reactions of nitro compounds with ylide (**2**) [23,39], it has been determined that the reaction between nitrodiene (**1**) and ylide (**2**) was performed via reflux in dry benzene. The molar ratio of main reagents was nitrodiene (**1**)–sarcosine (**9**)–formaldehyde (**10**) 1:20:30. The much bigger molar excess of compounds (**9**) and (**10**) necessary for N-methyl azomethine ylide (**2**) generation was used in order to enable the cycloaddition on both double bonds in (1*E*,3*E*)-1,4-dinitro-1,3-butadiene (**1**). The reaction progress was monitored by the TLC technique. On this basis, it was found that the conversion of substrates occurred in about 90 min. Furthermore, TLC analysis indicated that only one product was formed during the reaction. 

The obtained compound was isolated from the reaction mixture via column chromatography and then purified by crystallization. The final reaction product in the form of yellow crystals was identified based on physicochemical and spectral characteristics.

#### 2.2.3. Spectral Characteristics of the Obtained Product

In order to confirm the structure of the obtained compound, spectral physicochemical analyses as well as spectral characteristics such as HR-MS, IR, ^1^H NMR, ^13^C NMR and also 2D ^1^H-^13^C HMQC NMR were performed. The full spectral characteristics are included as Figures S1–S5 in the supporting information.

After isolation and purification of the obtained product, its melting point was measured at 91.6 °C. To confirm the structure of the synthesized compound, firstly, elemental analysis was performed. Based on the obtained results (Table 2), it was found that the percentage chemical composition of the synthesized compound does not correspond to any of the proposed products of single or double cycloaddition, compounds **3** and **4** in Scheme 1, respectively. 

**Scheme 4:**
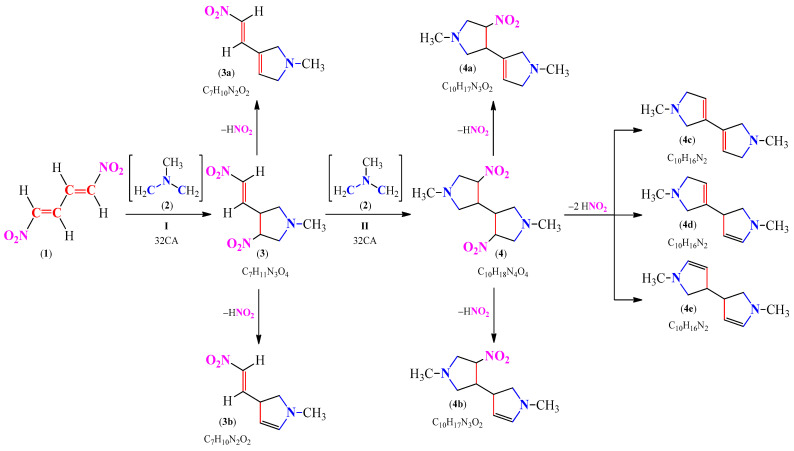
Theoretically possible reaction paths of 32CA between nitrodiene (**1**) and ylide (**2**), including the potential HNO_2_ elimination from the formed cycloadducts (**3**) and (**4**).

On this basis, it was assumed that the obtained compound had to undergo additional conversion. Therefore, it was decided to carry out an analysis of the newest literature discussing five-membered heterocyclic compounds containing a nitro group, with particular emphasis on bis-connection. Based on the similar cycloaddition reactions, it was noticed that these types of cycloadducts easily undergo the elimination reaction of the nitrous acid molecule from heterocyclic rings [39,40,67,68,69]. Thus, it was decided to take this aspect into account in further considerations. So, in a case of HNO_2_ elimination from 1-methyl-3-nitrovinyl-4-nitro-pyrrolidine (**3**), theoretically, two isomeric reaction products (**3a**) and (**3b**) are possible, while 1,1′-dimethyl-4,4′-dinitro-3,3′-bis-pyrrolidine (**4**) may have one HNO_2_ molecule removed (two isomeric alternatives (**4a**) and (**4b**) are possible), or it is possible for both HNO_2_ molecules to be removed (three isomeric alternatives (**4c**), (**4d**) and (**4e**) are possible) (Scheme 4). 

For theoretically possible isomers (**3a**,**b**) and (**4a**–**e**), obtained from cycloadducts **3** and **4** via HNO_2_ elimination from heterocyclic rings, the percentage chemical composition was calculated. Elemental analysis data gave the overall formula C_7_H_10_N_2_O_2_ for the isolated compound. Based on received information, it should be noted that the obtained values from the elemental analysis are compatible with structures of products (**3a**) and (**4a**) (Table 2 and Scheme 4). Additionally, for synthesized products, a high-resolution mass spectrum, obtained by an atmospheric pressure chemical ionization technique, was successfully performed. The HR-MS spectrum (Figure S1) is characterized by deprotonated molecular ions in negative mode [M-H]^−^. The elemental composition of the deprotonated molecular ion with *m*/*z* 153.0663 in negative mode confirmed the molecular formula C_7_H_10_N_2_O_2_. Thus, it should be concluded that the obtained compound will be isomer (**3a**) or (**3b**).

Important information could also be obtained from the analysis of the IR spectrum (Figure S2). In particular, in this spectrum, it was possible to characterize two intensive signals which were detected for the nitro group (stretch asymmetrical signal 1476 cm^−1^ and also stretch symmetrical signal 1322 cm^−1^).

Neither HR-MS nor IR analysis allows us to answer which of the two possible isomers, (**3a**) and (**3b**), was obtained in the studied reaction. Therefore, it was decided to perform NMR analysis including ^1^H NMR (Figure S3), ^13^C NMR (Figure S4) and 2D ^1^H-^13^C HMQC NMR (Figure S5) spectra. Thus, the distribution of signals in the ^1^H NMR spectrum allowed us to conclude that the synthesized compound is 1-methyl-3-(2-nitrovinyl-Δ^3^-pyrroline (**3a**) (Scheme 4). What is more, since the values of coupling constants *J* for doublets come from nitrovinyl moiety, it should be noted that the obtained Δ^3^-pyrroline (**3a**) is in *trans* configuration (CHα_NO2_, *J* = 13.3 Hz and CHα_NO2_, *J* = 13.4 Hz in the Supplementary Materials). On the other hand, thanks to 2D ^1^H-^13^C HMQC NMR analysis together with ^1^H NMR and also ^13^C NMR, it was possible to assign signals to all atoms in molecule (**3a**). Structural details are available in the Supplementary Materials.

Based on spectral characteristics, it should be concluded that in the reaction between (1*E*,3*E*)-1,4-dinitro-1,3-butadiene (**1**) and N-methyl azomethine ylide (**2**), only one reaction product is formed, which is 1-methyl-3-(*trans*-2-nitrovinyl)-Δ^3^-pyrroline (**3a**). Unfortunately, all attempts to obtain theoretically possible bis-adducts (**4** and **4a**–**e**) in laboratory conditions were unsuccessful.

### 2.3. Quantum-Chemical Structural Analysis of Pyrrolidine (***3***) and Δ^3^-Pyrroline (***3a***)

An extremely interesting observation of the experimental studies is the fact that despite the several dozen molar excess of N-methyl azomethine ylide (**2**) in relation to (1*E*,3*E*)-1,4-dinitro-1,3-butadiene (**1**), the cycloaddition occurs only on one of two available vinyl moieties of the nitrodiene (**1**). To explain this phenomenon, it was decided to carry out studies based on quantum-chemical calculation. 

#### 2.3.1. Electronic Properties of 1-Methyl-3-nitrovinyl-4-nitro-pyrrolidine (**3**)

Examples of double cycloaddition to CNDs are practically absent in the literature. In 2015, *Sharko* et al. [40] presented experimental studies of the reaction between (2*E*,4*E*)-2,5-dinitro-2,4-hexadiene and diazomethane. This reaction occurs on both double bonds of the tested diene. In the considered reaction of (1*E*,3*E*)-1,4-dinitro-1,3-butadiene (**1**) with N-methyl azomethine ylide (**2**), despite the use of a several dozen molar excess of the ylide (**2**), the reaction occurs only on the one of two available vinyl moieties of the nitrodiene (**1**). To explain this phenomenon, a thorough analysis of electronic properties of 1-methyl-3-nitrovinyl-4-nitro-pyrrolidine (**3**) based on MEDT [41] was carried out. 

**Figure 8:**
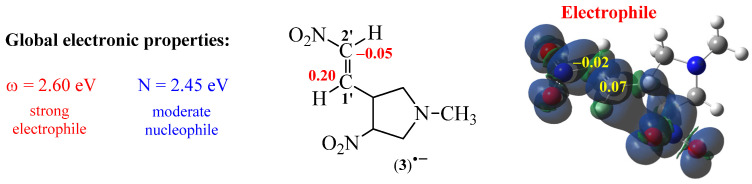
B3LYP/6-31G(d) global electrophilicity and global nucleophilicity indices, together with local electronic properties for pyrrolidine (**3**), presented as three-dimensional (3D) representations of Mulliken atomic spin densities for radical anion of **3^−^** and electrophilic P_k_^+^ Parr functions values (given in yellow), as well as the indices of the local electrophilicity ω_k_ of **3^−^** (given in red, in eV).

In the first part of the computational consideration, an analysis of reactivity indices for 1-methyl-3-nitrovinyl-4-nitro-pyrrolidine (**3**) according to the protocol of the CDFT was conducted. The global reactivity indices for pyrrolidine (**3**), together with the local electronic properties, are shown in Figure 8.

The calculated global electrophilicity [64] index ω of 1-methyl-3-nitrovinyl-4-nitro-pyrrolidine (**3**) is 2.60 eV, and the calculated global nucleophilicity [65] index N for this compound (**3**) is 2.45 eV (Figure 8). These values lead to the conclusion that the pyrrolidine (**3**) can be classified as a strong electrophile as well as a moderate nucleophile in polar reactions, within the electrophilicity and nucleophilicity scale [53,66]. Based on this information, it should be noted that in comparison to the global electronic properties of (1*E*,3*E*)-1,4-dinitro-1,3-butadiene (**1**), the computed value of the global electrophilicity of pyrrolidine (**3**) is significantly reduced, while the computed value of the global nucleophilicity of the compound (**3**) is significantly increased (Table 1). However, taking into account the values of global electronic properties of N-methyl azomethine ylide (**2**), it should be concluded that due to the extremely high computed value of the global nucleophilicity of the ylide (**2**) (N = 5.18 eV, Table 1), 1-methyl-3-nitrovinyl-4-nitro-pyrrolidine (**3**) can participate as an electrophilic component in a reaction with the ylide (**2**) [70]. However, in a reaction with N-methyl azomethine ylide (**2**), the pyrrolidine (**3**) is characterized by much lower electrophilic activity compared to nitrodiene (**1**).

The observations above are also confirmed by the analysis of local electronic properties of the pyrrolidine (**3**). In particular, the analysis indicates that the local electrophilicity ω_k_ indices for C1′ and C2′ carbon atoms of nitrovinyl moiety of the 1-methyl-3-nitrovinyl-4-nitro-pyrrolidine (**3**) are significantly reduced compared to analogous atoms in (1*E*,3*E*)-1,4-dinitro-1,3-butadiene (**1**). For the C1′ carbon atom of pyrrolidine (**3**), the value of the local electrophilicity index ω_k_ is ω_C1′_ = 0.20 eV (Figure 8). The values of the local electrophilicity index ω_k_ for the analogous carbon atoms in nitrodiene (**1**) are more than twice as high (ω_C_ = 0.42 eV, Figure 7). However, a much higher reduction is observed for the carbon atom directly connected with the nitro group. For the C2′ carbon atom of pyrrolidine (**3**), the value of the local electrophilicity index ω_k_ is ω_C1′_ = −0.05 eV (Figure 8), while the values of the local electrophilicity index ω_k_ for the analogous carbon atoms in nitrodiene (**1**) are ω_C_ = 0.64 eV (Figure 7). Based on the presented comparison of reactivity of pyrrolidine (**3**) with nitrodiene (**1**), it should be note that while the C1′ carbon atom shows slightly electrophilic activity, the C2′ carbon atom is a practically inactive electrophilic centre [71].

**Figure 9:**
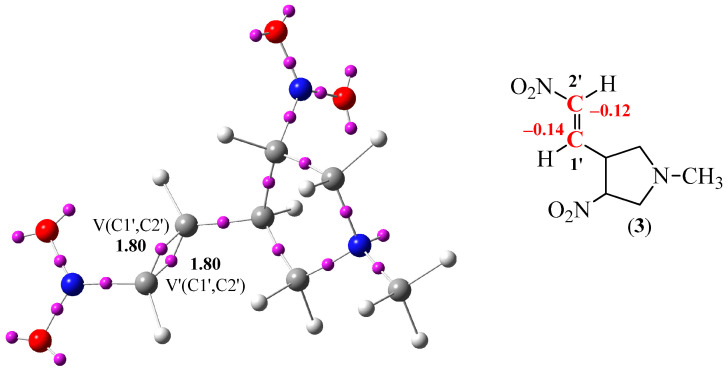
B3LYP/6-31G(d) ELF attractor positions of the valence basins with the most significant ELF valence basin populations and proposed ELF-based Lewis-like structures with the natural atomic charges for pyrrolidine (**3**). The ELF attractors are shown as pink spheres, while negative charges in Lewis-like structures are coloured in red. The electron populations as well as natural atomic charges are given as the average number of electrons [e].

In order to better understand the electronic structures of 1-methyl-3-nitrovinyl-4-nitro-pyrrolidine (**3**), an analysis of ELF together with NPA was performed (Figure 9).

The ELF topological analysis of the most significant C1′ and C2′ centres in the vinyl fragment of the pyrrolidine (**3**) shows very similar results to nitrodiene (**1**). In particular, the presence of two pairs of disynaptic basins, namely V(C1′,C2′) and V’(C1′,C2′), integrating a total electron population of 3.60 e, is observed (Figure 9), which is a value only slightly higher than in the case of nitrodiene (**1**) (3.46 e, Figure 2). The presence of these disynaptic basins is associated with a somewhat depopulated C1′-C2′ double bond, but still not more so than in nitrodiene (**1**). This may be related to a uniquely stable double-bond system.

In turn, the NPA analysis of the centres most significant for the vinyl fragment for pyrrolidine (**3**), C1′ and C2′, indicates a different distribution of the natural atomic charges compared to nitrodiene (**1**). While for (1*E*,3*E*)-1,4-dinitro-1,3-butadiene (**1**), a significant difference between carbon atoms in vinyl fragment (around 0.10 e, Figure 4) can be observed, for 1-methyl-3-nitrovinyl-4-nitro-pyrrolidine (**3**), the difference is practically non-existent (Figure 9).

**Figure 10:**
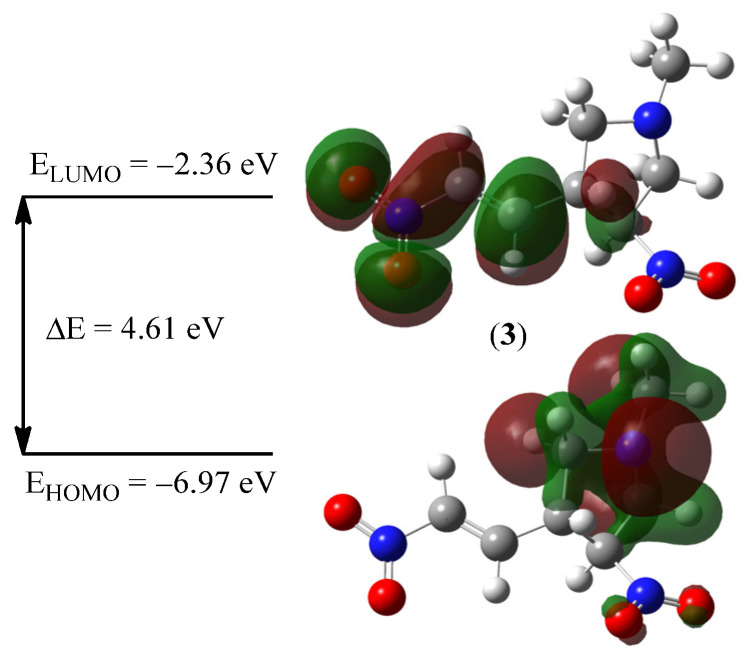
B3LYP/6-31G(d) HOMO–LUMO energy gap ΔE diagram for pyrrolidine (**3**).

And finally, in order to confirm the lower reactivity of 1-methyl-3-nitrovinyl-4-nitro-pyrrolidine (**3**) towards (1*E*,3*E*)-1,4-dinitro-1,3-butadiene (**1**), the HOMO–LUMO energy gap was calculated (Figure 10). The computed HOMO energy of pyrrolidine (**3**) is −6.97 eV, and the computed LUMO energy for this compound (**3**) is −2.36 eV (Figure 10). Consequently, the HOMO–LUMO energy gap ΔE is 4.61 eV, and this value is higher than nitrodiene (**1**) (4.40, Figure 5). As mentioned earlier, the HOMO–LUMO energy gap is an important stability index. A molecule with a high frontier molecular orbital HOMO–LUMO energy gap ΔE is characterized by low chemical reactivity and simultaneously high kinetic stability.

The theoretical premises indicate that 1-methyl-3-nitrovinyl-4-nitro-pyrrolidine (**3**) will not undergo further cycloaddition at the second vinyl moiety; experimental reports also support this argument. The experimentally observed and confirmed phenomenon can be explained easily on the basis of a DFT study. It is known that the presence of alkylamino groups at the 2 position of the nitrovinyl moiety drastically stimulates its activity. This was recently confirmed based on the comprehensive theoretical and experimental studies [72].

#### 2.3.2. Analysis of the Structural and Stability Aspects of Possible Forms of 1-Methyl-3-(*trans*-2-nitrovinyl)-Δ^3^-pyrroline (**3a**)

**Figure 11:**
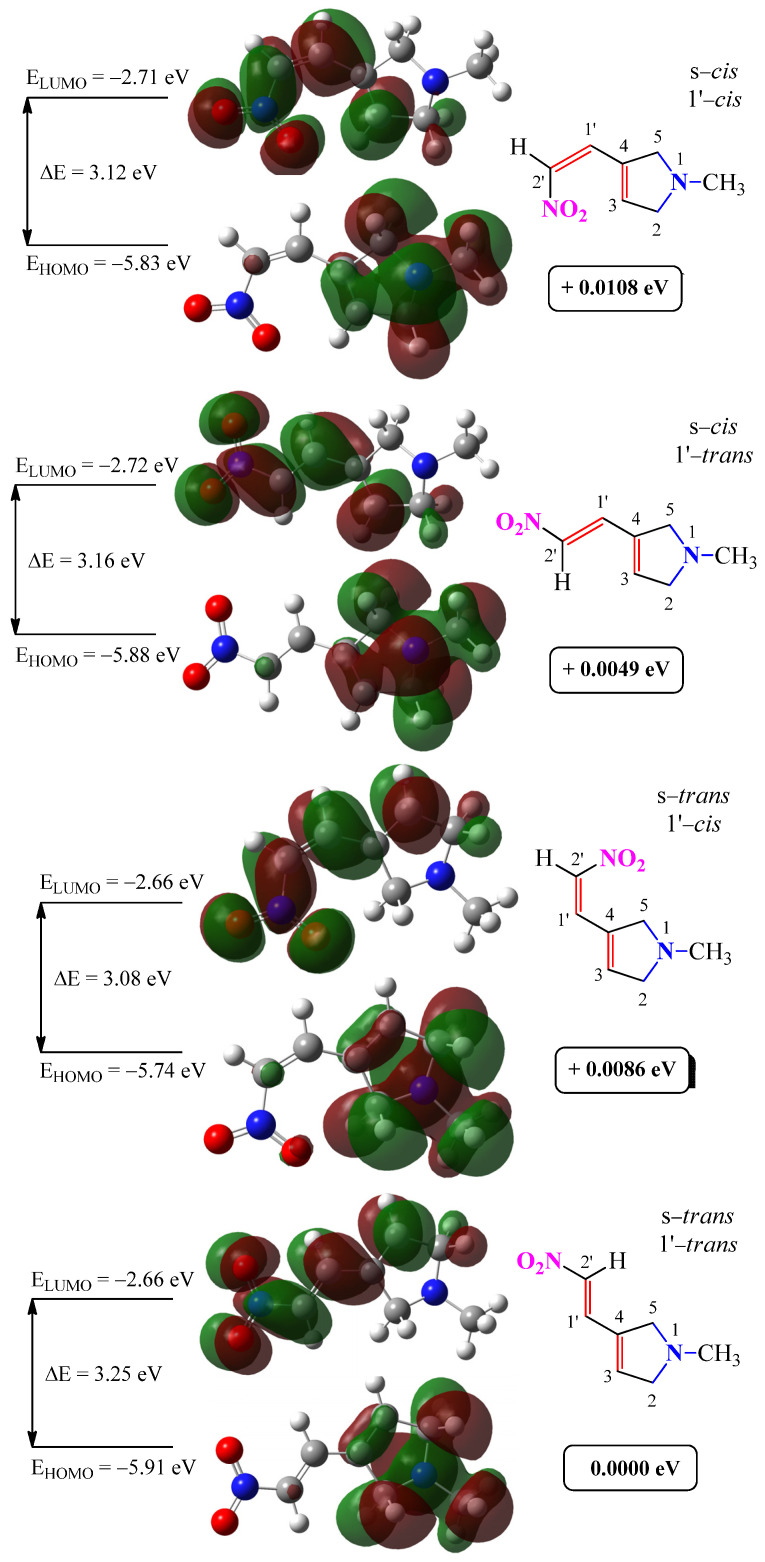
B3LYP/6-31G(d) HOMO–LUMO energy gap ΔE diagram together with values of the relative global minimums, given in frame, for theoretical possible conformers of Δ^3^-pyrroline (**3a**).

**Figure 12:**
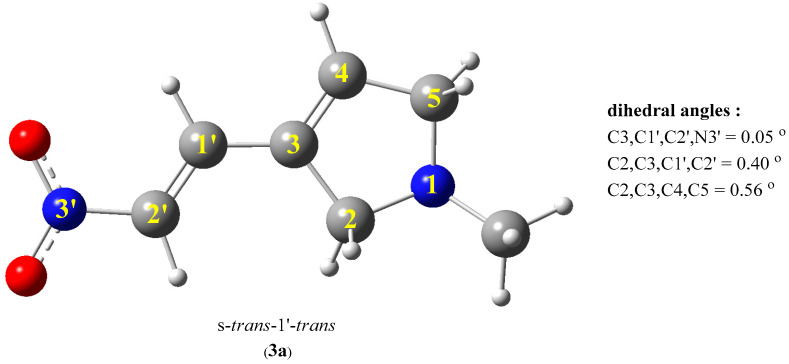
B3LYP/6-31G(d) computed structure of s-*trans*-1′-*trans* conformer of pyrrolidine (**3**).

In the next part, the key structural aspects for the obtained 1-methyl-3-(*trans*-2-nitrovinyl)-Δ^3^-pyrroline (**3a**) were investigated. For this purpose, the HOMO–LUMO energy gap diagram (Figure 11) as well as the most important dihedral angles (Figure 12) of Δ^3^-pyrroline (**3a**) were analysed. 

Theoretically, the 1-methyl-3-(2-nitrovinyl)-Δ^3^-pyrroline (**3a**), can exist in four conformational forms (Figure 11). Two structures, characterized by *cis* isomerism on the nitrovinyl segment, should be excluded based on the evident observation from NMR structural experiments (see 2.2.3). Therefore, only two structures, s-*cis*-1′-*trans* and s-*trans*-1′-*trans*, should be formally considered. Despite this fact, the analyses of the energetic stability were performed for all four molecules, in order to show that both s-*cis*-1′-*cis* and s-*trans*-1′-*cis* conformers are characterized by a higher relative global minimum and lower HOMO–LUMO energy gap in comparison to the two possible s-*cis*-1′-*trans* and s-*trans*-1′-*trans* forms.

On the other hand, among the other two s-*cis*-1′-*trans* and s-*trans*-1′-*trans* conformers, the s-*trans*-1′-*trans* conformer is characterized both by the highest relative global minimum as well as the lowest HOMO–LUMO energy gap (Figure 11). Both of these parameters indicate that it is the most stable of all the conformers.

For the most stable s-*trans*-1′-*trans* conformer of 1-methyl-3-(*trans*-2-nitrovinyl)-Δ^3^-pyrroline (**3a**), the most important dihedral angles were determined (Figure 12). Based on obtained results, it should be noted that key dihedral angles are approximately zero. This confirms the almost planar structure of the key segment of the analysed molecule.

### 2.4. In Silico Study of Biological Potential of Obtained 1-Methyl-3-(trans-2-nitrovinyl)-Δ^3^-pyrroline (***3a***) Based on ADME and PASS

#### 2.4.1. Analysis of Druglikeness and ADME Studies of Δ^3^-Pyrroline (**3a**)

Finally, for obtained 1-methyl-3-(*trans*-2-nitrovinyl)-Δ^3^-pyrroline (**3a**), a comprehensive evaluation of the potential biological activity was determined. In the literature, there are many examples showing that pyrroline derivatives exhibit biological activity [73,74,75]. In particular, the presence of a nitro group in the structure strongly enhances this effect [6,7,8]. To evaluate the potential biological activity, a simple and widely used in silico protocol of ADME [42] was applied. In order to underline a biological significance of 1-methyl-3-(*trans*-2-nitrovinyl)-Δ^3^-pyrroline (**3a**) and predict potential application paths for this compound (**3a**), a common protocol of PASS [43] was also carried out.

ADME studies are critical in modern drug discovery. The process of predicting a good drug by analysing its pharmacokinetic properties in the laboratory is expensive and labour intensive. So, to accelerate the research and more rationally dispense the financing, ADME parameters can be inspected using in silico tools [76]. In view of the above, selected physicochemical properties like lipophilicity, water solubility, pharmacokinetics and also medicinal chemistry friendliness for the synthesized compound were assessed. For this purpose, an online server SwissADME [77] was used. The most important information is collected in Table 3. 

In order to evaluate the druglikeness for obtained computational results, the assessment via models specified by *Lipinski* et al. [78], *Ghose* et al. [79], *Veber* et al. [80], *Egan* et al. [81] and *Muegge* et al. [82] was carried out. The main features and conditions of these rules and filters are collected in Table 4.

Based on selected physicochemical and biological parameters predicted for 1-methyl-3-(*trans*-2-nitrovinyl)-Δ^3^-pyrroline (**3a**) via ADME (Table 3), as well as the main features of the five druglikeness rules (Table 4), it should be concluded that the obtained Δ^3^-pyrroline (**3a**) is a good drug candidate according to the filters by *Lipinski* et al. [78], *Veber* et al. [80] and *Egan* et al. [81]. The synthesized compound (**3a**) is characterized by appropriate molecular weight, number of rotatable bonds, H-bond donor–acceptor ratio, as well as satisfactory value of topological polar surface area (TPSA), which is a commonly used parameter for the optimization of a drug’s ability to permeate cells [83]. What is more, the obtained Δ^3^-pyrroline (**3a**) can present desired lipophilicity properties.

On the other hand, based on more demanding filters such as those by *Ghose* et al. [79] and *Muegge* et al. [82], 1-methyl-3-(*trans*-2-nitrovinyl)-Δ^3^-pyrroline (**3a**) is characterized as having insufficient potential as a drug candidate. Both of the filters define a minimum molecular weight, which is the only one problem for structure (**3a**). 

The assessment of other important druglikeness parameters for 1-methyl-3-(*trans*-2-nitrovinyl)-Δ^3^-pyrroline (**3a**) shows that the synthesized compound (**3a**) is characterized not only by promising lipophilic properties but is also water-soluble (Table 3). These physicochemical properties are crucial to drugs’ uptake and their further metabolism [74]. 

In terms of medicinal chemistry friendliness, Δ^3^-pyrroline (**3a**) is negative Pan Assay Interference Compounds (PAINS) and causes only one alert, which is related with the presence of a nitro group in its structure (Table 3). However, according to the literature [6,7,8], it should be underlined that in many examples, the presence of a nitro group in the structure of conjugated compounds additionally stimulates a biological activity.

Additionally, it should be concluded that 1-methyl-3-(*trans*-2-nitrovinyl)-Δ^3^-pyrroline (**3a**) can have good gastrointestinal (GI) absorption (Table 3). What is more, according to the performed simulation, the synthesized compound (**3a**) inhibits only one of the tested cytochrome P450 isoforms, which is CYP1A2 (Table 3).

**Figure 13:**
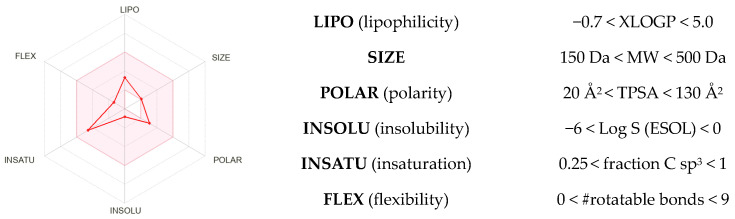
The bioavailability radar for 1-methyl-3-(*trans*-2-nitrovinyl)-Δ^3^-pyrroline (**3a**). The pink area represents the optimal range for each property, including lipophilicity, size, polarity, insolubility, insaturation and flexibility.

Finally, the bioavailability radar, which allows us to visually assess the druglikeness of compounds, was evaluated. Based on analysis of the bioavailability radar for 1-methyl-3-(*trans*-2-nitrovinyl)-Δ^3^-pyrroline (**3a**), it should be concluded that the only problem is the too low molecular weight of the obtained compound (**3a**). In particular, the MW is at the lower limit of the radar (Figure 13). 

#### 2.4.2. Assessment of Antimicrobial Activities and Potential Biological Application of Δ^3^-Pyrroline (**3a**) Based on PASS

The potential biological application for synthesized Δ^3^-pyrroline (**3a**) was investigated via PASS [84]. The PASS is a simple and very useful tool to allow preliminarily assessment of a molecule’s druglikeness. The results of PASS analysis are expressed as the molecule’s probability of being active (Pa) or inactive (Pi). The values of Pa and Pi range from 0.000 to 1.000, where the value of 0.000 means a complete lack of probability, while a value of 1.000 means certainty. The compound can be assigned as probably active when Pa > Pi. The obtained data are useful to decide if the synthesis and characterization of novel compounds is justified prior to preparing them in the laboratory and if the screened compounds are good candidates for further biological research [43]. In order to evaluate potential antimicrobial properties of 1-methyl-3-(*trans*-2-nitrovinyl)-Δ^3^-pyrroline (**3a**), an assessment of selected activities was performed by PASS [84]. The obtained results are collected in Table 5.

According to the PASS analysis, the 1-methyl-3-(*trans*-2-nitrovinyl)-Δ^3^-pyrroline (**3a**) is characterized by a very low potential of antimicrobial activity. In particular, all Pa parameters against microorganisms, namely antiviral, antifungal, antibacterial, as well as antiparasitic, are lower than 0.350. To deem a compound potentially active, the Pa parameter should be higher than 0.700 [43]. 

In connection with this, it was decided to determine the biological activities of 1-methyl-3-(*trans*-2-nitrovinyl)-Δ^3^-pyrroline (**3a**) for which the Pa index is higher than the reference value 0.700 [43]. The obtained results are collected in Table 6.

According to the PASS analysis, for the 1-methyl-3-(*trans*-2-nitrovinyl)-Δ^3^-pyrroline (**3a**), only a few potential application directions are available. The most promising includes nicotinic alpha-6-beta-3-beta-4-alpha-5 receptor antagonist as well as (R)-6-hydroxynicotine oxidase inhibitor (Table 6). However, it should be underlined that the Pa values for both proposed applications are close to the lower limit of the reference value of Pa [43]. Therefore, to confirm for this hypothesis in the future, in vitro and/or in vivo studies would be necessary.

## 3. Materials and Methods

### 3.1. Materials

Commercially available (Sigma–Aldrich, Szelągowska 30, 61-626 Poznań, Poland) reagents and solvents were used. All solvents were tested with high-pressure liquid chromatography before use.

### 3.2. Synthesis of Nitrodiene (***1***) and Ylide (***2***)

The components needed for the reaction were prepared according to well-known and available procedures described in the literature. In particular, the (1*E*,3*E*)-1,4-dinitro-1,3-butadiene (**1**) was synthesized via a three-step protocol, starting from nitromethane (**5**) and glyoxal (**6**) [37]. In turn, the N-methyl azomethine ylide (**2**) was generated *in situ* from sarcosine (**9**) and formaldehyde (**10**) in a form of paraformaldehyde (**10′**) [23,39]. 

### 3.3. Cycloaddition Between Nitrodiene (***1***) and Ylide (***2***)—General Procedure

To a 100 mL flask equipped with a reflux condenser, a magnetic stirrer and a heating mantle was added. To the flask, 3.5 mmol of 1,4-dinitrobuta-1,3-diene (**1**), 20 mmol of sarcosine (**9**), 115 mmol of paraformaldehyde (**10′**) and 50 mL of dry benzene were added. The reagents were heated together under reflux for 90 min. After that, the post-reaction mixture was cooled and filtered. The obtained sediment was washed twice with 20 mL of benzene. Then, the filtrate was evaporated in a vacuum evaporator (40 °C). The dry residue was separated chromatographically on a column filled with silica gel via a mixture of cyclohexane–ethyl acetate CyH:EtOAc (50:50 *v*/*v*). The obtained product was purified by crystallization from diethyl ether. Finally, the 1-methyl-3-(*trans*-2-nitrovinyl)-Δ^3^-pyrroline (**3a**) was obtained in the form of yellow crystals.

1-methyl-3-(*trans*-2-nitrovinyl)-Δ^3^-pyrroline (**3a**): m.p. 91.6 °C; FT-IR (ATR): υ [cm^−1^] 3114 (~C–H stretch, alkene, medium), 2917 (~C–H stretch, alkane, medium), 1727 (>C=C< stretch, *trans* alkene, weak), 1614 (C=C< stretch, conjugated alkene, strong), 1537 (~C–H bend, alkene, medium), 1476 (~N–O stretch, asymmetrical, nitro group, strong), 1420 (~CH_3_ rock, medium), 1376 (~CH_3_ rock, medium), 1322 (~N–O stretch, symmetrical, nitro group, strong), 1232 (–N< stretch, pyrroline ring, medium), 1154 (–N< stretch, pyrroline ring, medium), 955 (>C=C< bend, *trans* alkene, strong), 717 (=C–H bend, alkene, strong); ^1^H NMR (400 MHz, CDCl_3_): δ [ppm] 7.96 (d, 1H, C**H**–NO_2_, J = 13.3 Hz), 7.39 (d, 1H, =C**H**–ring, J = 13.4 Hz), 7.03–7.01 (m, 1H, =C**H**–ring), 6.67–6.65 (m, 2H, =C**H**–ring), 6.38–6.36 (m, 2H, =C**H**–ring); ^13^C NMR (100 MHz, CDCl_3_): δ [ppm] 134.08, 132.82, 128.06, 125.08, 115.92, 107.71, 36.65; EA: calculated using the brutto formula C_7_H_10_N_2_O_2_ [%]: C 54.55, H 6.49, N 18.18, found, %: C 54.52, H 6.51, N 18.20. HR-MS: (−APCI): *m*/*z* calculated using the formula C_7_H_9_N_2_O_2_: 153.0664 [M-H]^−^; found 153.0663.

### 3.4. Analytical Techniques

For reaction progress testing, thin-layer chromatography (TLC) was performed using aluminium-backed silica plates (unmodified layers) as the standard procedure in a case of nitro-organic compounds [72,85]. In the role of eluent, cyclohexane–ethyl acetate mixture CyH:EtOAc (50:50 *v*/*v*) was applied. The plates were developed by iodine treatment. Melting points were determined with the Boetius PHMK 05 apparatus and were not corrected. Elemental analyses were performed on a Perkin-Elmer PE-2400 CHN apparatus. HRMS analysis was performed using a Synapt G2-Si mass spectrometer equipped with an atmospheric pressure chemical ionization (APCI) source and combined quadrupole time-of-flight (QTOF) mass analyser. The heated capillary temperature was 350 °C. To ensure accurate mass measurements, data were collected in centroid mode, and mass was corrected during acquisition using leucine enkephalin solution as an external reference (Lock-SprayTM). FT-IR analysis was derived from the FTS Nicolet IS 10 spectrophotometer with attenuated total reflectance (ATR). ^1^H NMR (400 MHz) and ^13^C NMR (100 MHz) analysis were recorded with a Bruker AVANCE NMR spectrometer. All spectra were obtained in the deuterated chloroform CDCl_3_ (visible at 7.27 ppm for ^1^H NMR and at 77.00 ppm for ^13^C NMR) solutions, and the chemical shifts (δ) are expressed in ppm, while the J-couplings (J) are given in Hz. TMS was used as an internal standard.

### 3.5. Computational Details

All computations were performed using the Gaussian 16 package [86] in the Ares computer cluster of the CYFRONET regional computer centre in Cracow. DFT calculations were performed using the B3LYP functional [87] together with the 6-31G(d) basis set [88]. This computational level is required by the MEDT approach [41] and has already been successfully used in optimization and evaluation of a various organic molecules, especially conjugated nitro-organic systems [89,90,91,92]. What is more, this computational level correlates well with experimental results [93,94,95,96]. Calculations of all critical structures were performed at temperature T = 298 K and pressure *p* = 1 atm in a gas phase. All localized stationary points were characterized using vibrational analysis. It was found that starting molecules as well as products had positive Hessian matrices. 

The electronic structures of (1*E*,3*E*)-1,4-dinitro-1,3-butadiene (**1**) and N-methyl azomethine ylide (**2***)* were characterized by the Electron Localization Function (ELF) [44], the Natural Population Analysis (NPA) [45,46], as well as the molecular electrostatic potential (MEP) [47]. Analyses of global electronic properties of reagents were performed according to *Domingo’s* recommendations [53,54,55]. Electrophilic Parr functions P_k_^+^ and nucleophilic Parr functions P_k_^−^ were obtained from the atomic spin density (ASD) of the reagents’ radical ions [56,57]. 

The physicochemical properties for the obtained compound were determined via the SwissADME online server [77]. In turn, the assessment of the druglikeness was performed based on models and rules of *Lipinski* et al. [78], *Ghose* et al. [79], *Veber* et al. [80], *Egan* et al. [81] and *Muegge* et al. [82]. On the other hand, the prediction of the main antimicrobial activities and also potential application routes for obtained compound were carried out via the PASS online server [84].

The NPA and the ELF studies were performed with TopMod 09 [97] software. For visualization of the molecular geometries of all structures and 3D representations of the radical anions and the radical cations, GaussView 6.0 software [98] was used. In turn, the ELF localization domains were represented by using the ParaView 5.9.1 software [99] at an isovalue of 0.75 a.u.

## 4. Conclusions and Future Prospects

In this research, a comprehensive experimental as well as quantum-chemical study of the cycloaddition reaction between (1*E*,3*E*)-1,4-dinitro-1,3-butadiene (**1**) and N-methyl azomethine ylide (**2**) was presented. Additionally, the study was enriched with simple predictions of the biological potential of the obtained structure.

The computational results based on Molecular Density Functional Theory confirm the conjugated character of the nitrodiene (**1**) and indicate an N-centred ally pseudodiradical structure of the tested ylide (**2**). Analysis of the reactivity for the presented reagents suggests that the nitrodiene (**1**) will participate as electrophilic agents, while the studied ylide (**2**) will pay a role of nucleophilic substances. And finally, the calculation indicates that the reaction will have an evidently polar character with the forward electron density flux from ylide (**2**) to diene (**1**) [59].

In turn, the experimental results for the cycloaddition between (1*E*,3*E*)-1,4-dinitro-1,3-butadiene (**1**) and N-methyl azomethine ylide (**2**) show that the reaction is realized on only one of the two available double bonds of nitrodiene (**1**). Additionally, from the formed heterocyclic ring, an HNO_2_ molecule is eliminated. As a result, the 1-methyl-3-(*trans*-2-nitrovinyl)-Δ^3^-pyrroline (**3a**) is created as the only reaction product. These observations are extremely interesting. In order to generate the ylide (**2**) in the tested reaction, several dozen molars were used compared to the nitrodiene (**1**). Nevertheless, the cycloaddition occurred only on one of the two available vinyl moieties of the nitrodiene (**1**). This observation is phenomenal because so far, no other similar example has been found in the literature [25,26,27,28]. Simultaneously, the tested reaction shows the instability of the nitro group directly located on the heterocyclic ring and confirms that in some cases, its elimination can occur [40,67,68,69]. 

All attempts to experimentally obtain theoretically possible bis-adducts (**4** and **4a**–**e**) were unsuccessful. This is due to significantly lower chemical activity and also higher kinetic stability of 1-methyl-3-nitrovinyl-4-nitro-pyrrolidine (**3**), as compared to (1*E*,3*E*)-1,4-dinitro-1,3-butadiene (**1**), in a reaction with N-methyl azomethine ylide (**2**). What is more, the formation of the 1-methyl-3-(*trans*-2-nitrovinyl)-Δ^3^-pyrroline (**3a**) instead of 1-methyl-4-(*trans*-2-nitrovinyl)-Δ^2^-pyrroline (**3b**) can also be explained based on a structural aspect of similar examples available in the literature [100]. In case of the compound (**3a**), a conjugated system of π-bonds is formed. This additionally stabilizes the molecule. In the case of a theoretically possible but not formed compound (**3b**), the isolated system of double bonds does not exist.

Additionally, it is worth mentioning that the less reactive diazomethane reacts with (2E,4E)-2,5-dinitrohexa-2,4-diene at both available bonds of the nitrodiene [40]. It is surprising that the more reactive N-methyl azomethine ylide (**2**) reacts only at one bond even though it is introduced into the reaction in a large molar excess. The TAC type is probably responsible for the difference in reactivity. While diazomethane shows *pmr*-type reactivity, the tested in this research ylide has a *pdr*-type electronic structure [38,51].

The structure of the obtained 1-methyl-3-(*trans*-2-nitrovinyl)-Δ^3^-pyrroline (**3a**) has been confirmed based on spectral analyses such as HR-MS, IR, ^1^H NMR, ^13^C NMR and 2D ^1^H-^13^C HMQC NMR.

According to basic analysis of physicochemical descriptors as well as predicted ADME and PASS parameters, it can be concluded that the 1-methyl-3-(*trans*-2-nitrovinyl)-Δ^3^-pyrroline (**3a**) can demonstrate a potential biological activity. Both the applied filters [75,76,77,78,79] and the bioavailability radar analysis indicate that Δ^3^-pyrroline (**3a**) may have too low a molecular weight. However, according to the PASS analysis for the Δ^3^-pyrroline (**3a**), too few potential directions of potential biological applications are available.

Taking into account the unexpected structure of the final product, the obtained 1-methyl-3-(*trans*-2-nitrovinyl)-Δ^3^-pyrroline (**3a**) constitutes an interesting compound for further transformations. Due to presence of active nitrovinyl moiety as well as an unsaturated bond in the ring in the structure of 1-methyl-3-(*trans-*2-nitrovinyl)-Δ^3^-pyrroline (**3a**), future modification of this compound (**3a**) should be simple to perform. The transformation of molecule (**3a**) into a compound with higher molecular weight can provide greater probability of biological activity occurrence. 

## Figures and Tables

**Table 1:** B3LYP/6-31G(d) HOMO and LUMO energies as well as global reactivity indices for nitrodiene (**1**) and ylide (**2**), given in electronvolts [eV].

[eV]	1	2
HOMO energy	−8.31	−3.94
LUMO energy	−3.91	0.35
Energy gap, ΔE	4.40	4.49
Electronic chemical potential, μ	−6.11	−1.79
Chemical hardness, η	4.40	4.29
Chemical softness, S	0.23	0.23
Global electrophilicity, ω	4.24	0.38
Global nucleophilicity, N	0.81	5.18

**Table 2:** The results of elemental analysis for the obtained product of the reaction between nitrodiene (**1**) and ylide (**2**).

	Hypothetical Product (3)	Hypothetical Product (4)	Obtained Results
%C	41.79%	46.51%	54.52%
%H	5.47%	7.00%	6.51%
%N	20.90%	21.71%	18.20%

**Table 3:** Druglikeness parameters for 1-methyl-3-(*trans*-2-nitrovinyl)-Δ^3^-pyrroline (**3a**).

Physicochemical Properties												
Formula						C_7_H_10_N_2_O_2_						
Molecular weight MW						154.17 g/mol						
#Heavy atoms						11						
#Aromatic heavy atoms						0						
#Rotatable bonds						2						
#H-bond acceptors						3						
#H-bond donors						0						
Molar refractivity MR						47.62						
Topological polar surface area TPSA						49.06 Å^2^						
**Lipophilicity Log P_o/w_**												
iLOGP		XLOGP		WLOGP		MLOGP			SILICOS-IT		Consensus	
1.63		0.30		0.79		−0.32			−0.66		0.35	
**Water Solubility Log S**												
Log S (ESOL)		Solubility		Class		Log S (Ali)			Solubility		Class	
−0.85		21.6 mg/mL		Very soluble		−0.89			19.7 mg/mL		Very soluble	
**Pharmacokinetics**												
IG absorption	BBB permeant		CYP1A2 INH		CYP2C19 INH		CYP2C9 INH	CYP2D6 INH		CYP3A4 INH		Log K_p_ Skin permeation
High	Yes		Yes		No		No	No		No		−7.03 cm/s
**Medicinal Chemistry Friendliness**												
PAINS				Brenk					Synthetic accessibility			
0 alerts				1 alert					31.7%			

**Table 4:** Main features of the five druglikeness rules evaluated in this study.

*Lipinski* et al. [75] (Pfizer)	*Ghose* et al. [76] (Amgen)	*Veber* et al. [77] (GSK)	*Egan* et al. [78] (Pharmacia)	*Muegge* et al. [79] (Bayer)
MW ≤ 500 Da MLOGP ≤ 4.15 #H-bond donors ≤ 5 #H-bond acceptors ≤ 10	160 Da ≤ MW ≤ 480 Da −0.4 ≤ WLOGP ≤ 5.6 40 ≤ MR ≤ 130 20 ≤ #atoms ≤ 70	#Rotatable bonds ≤ 10 TPSA ≤ 140 Å^2^	WLOGP ≤ 5.88 TPSA ≤ 131.6 Å^2^	200 Da ≤ MW ≤ 600 Da −0.4 ≤ XLOGP ≤ 5.6 TPSA ≤ 150 Å^2^ #Rings ≤ 7 #Carbons > 4 #Heteroatoms > 1 #Rotatable bonds ≤ 15 #H-bond donors ≤ 5 #H-bond acceptors ≤ 10

**Table 5:** PASS prediction of the main antimicrobial activities for 1-methyl-3-(*trans*-2-nitrovinyl)-Δ^3^-pyrroline (**3a**). The results are expressed as a molecule’s probability of being active (Pa) or inactive (Pi).

Antimicrobial Activity	Pa	Pi
Antiviral (Adenovirus)	0.340	0.060
Antiviral (Picornavirus)	0.333	0.179
Antifungal	0.281	0.090
Antibacterial	0.237	0.090
Antiparasitic	0.143	0.136

**Table 6:** The PASS prediction of the main potential activities of the 1-methyl-3-(trans-2-nitrovinyl)-Δ^3^-pyrroline (**3a**). The results are expressed for a molecule’s probability of being active Pa > 0.700.

Biological Activity	Pa	Pi
Nicotinic alpha-6-beta-3-beta-4-alpha-5 receptor antagonist	0.742	0.023
(*R*)-6-hydroxynicotine oxidase inhibitor	0.724	0.006

## Data Availability

The data presented in this study are available on request from the corresponding author.

## Supplementary Materials

The following supporting information can be downloaded at: https://www.mdpi.com/article/10.3390/molecules29215066/s1, experimental details and spectra: pp. S2–S4; computational data: pp. S5.
