# Supplementary material for: Unexpected Course of Reaction Between (1E,3E)-1,4-Dinitro-1,3-butadiene and N-Methyl Azomethine Ylide—A Comprehensive Experimental and Quantum-Chemical Study
Source: Molecules. 2024 Oct 26;29(21):5066. doi: 10.3390/molecules29215066 (PMC11547607; doi:10.3390/molecules29215066)
Supplement: Supplementary file 1 [file molecules-29-05066-s001.zip › molecules-3244651-supplementary.pdf]

SUPPLEMENTARY MATERIALS

Unexpected course of reaction between (1E,3E)-1,4-dinitro-1,3-butadiene and N-methyl azomethine ylide — A comprehensive experimental and quantum-chemical study

Mikołaj Sadowski and Karolina Kula *

Department of Organic Chemistry and Technology, Cracow University of Technology, Warszawska 24, 31-155 Cracow, Poland;
*Correspondence adress: karolina.kula@pk.edu.pl (K.K)

PHYSICAL CHARACTERISTICS

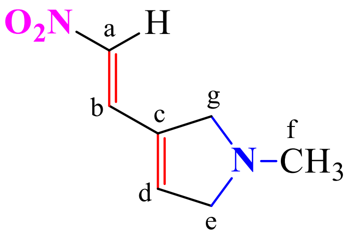<br>(**3a**)<br>$C_7H_{10}N_2O_2$	1-methyl-3-(*trans*-2-nitrovinyl)-Δ[3]-pyrroline (**3a**):<br>Yellow crystal solid, m.p. 91.6 °C (diethyl ether)<br><br>EA:<br>Calculated for brutto formula $C_7H_{10}N_2O_2$ [%]: C 54.55, H 6.49, N 18.18; Found [%]: C 54.52, H 6.51, N 18.20.<br><br>HR-MS: (−APCI): m/z<br>Calculated for formula $C_7H_9N_2O_2$: 153.0664 $[M\text{-}H]^-$; Found 153.0663<br><br>FT-IR (ATR): υ [cm^{-1}]<br>3114 (~C–H stretch, alkene, medium),<br>2917 (~C–H stretch, alkane, medium),<br>1727 (>C=C< stretch, *trans* alkene, weak),<br>1614 (C=C< stretch, conjugated alkene, strong),<br>1537 (~C–H bend, alkene, medium),<br>1476 (~N–O stretch, asymmetrical, nitro group, strong),<br>1420 (~CH_3 rock, medium),<br>1376 (~CH_3 rock, medium),<br>1322 (~N–O stretch, symmetrical, nitro group, strong),<br>1232 (–N< stretch, pyrroline ring, medium),<br>1154 (–N< stretch, pyrroline ring, medium),<br>955 (>C=C< bend, *trans* alkene, strong),<br>717 (=C–H bend, alkene, strong).<br><br>^1H NMR (400 MHz, $CDCl_3$): δ [ppm]<br>7.96 (d, 1H, $C\mathbf{H}_a$–NO_2, J = 13.3 Hz),<br>7.39 (d, 1H, =$C\mathbf{H}_b$–ring, J = 13.4 Hz),<br>7.03 – 7.01 (m, 1H, =$C\mathbf{H}_d$–ring),<br>6.67 – 6.65 (m, 2H, =$C\mathbf{H}_g$–ring),<br>6.38 – 6.36 (m, 2H, =$C\mathbf{H}_e$–ring).<br><br>^{13}C NMR (100 MHz, $CDCl_3$): δ [ppm]<br>134.08 ($\mathbf{C}_a$), 132.82 ($\mathbf{C}_b$), 128.06 ($\mathbf{C}_d$), 125.08 ($\mathbf{C}_g$), 115.92 ($\mathbf{C}_c$), 107.71 ($\mathbf{C}_e$), 36.65 ($\mathbf{C}_f$).

HR-MS

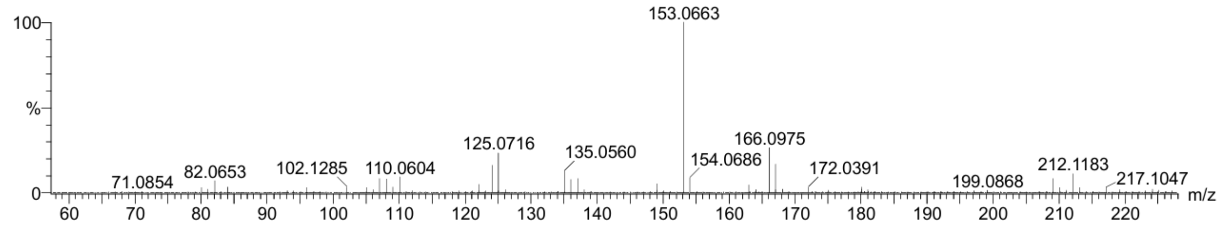

Minimum:				-1.5				
Maximum:		5.0	5.0	90.0				
Mass	Calc. Mass	mDa	PPM	DBE	i-FIT	Norm	Conf(%)	Formula
153.0663	153.0664	-0.1	-0.7	4.5	1253.1	n/a	n/a	C7 H9 N2 O2

Figure S1. HR-MS spectrum of 1-methyl-3-(*trans*-2-nitrovinyl)-Δ^3-pyrroline (**3a**).

FT-IR

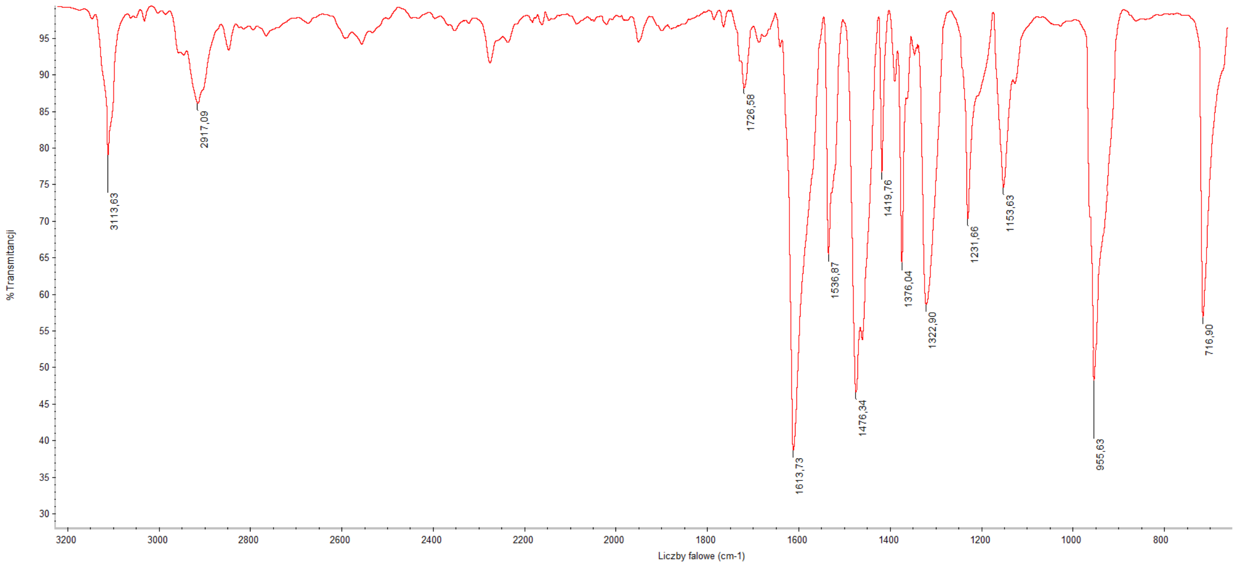

Figure S2. FT-IR spectrum of 1-methyl-3-(*trans*-2-nitrovinyl)-Δ^3-pyrroline (**3a**).

^{1}H NMR

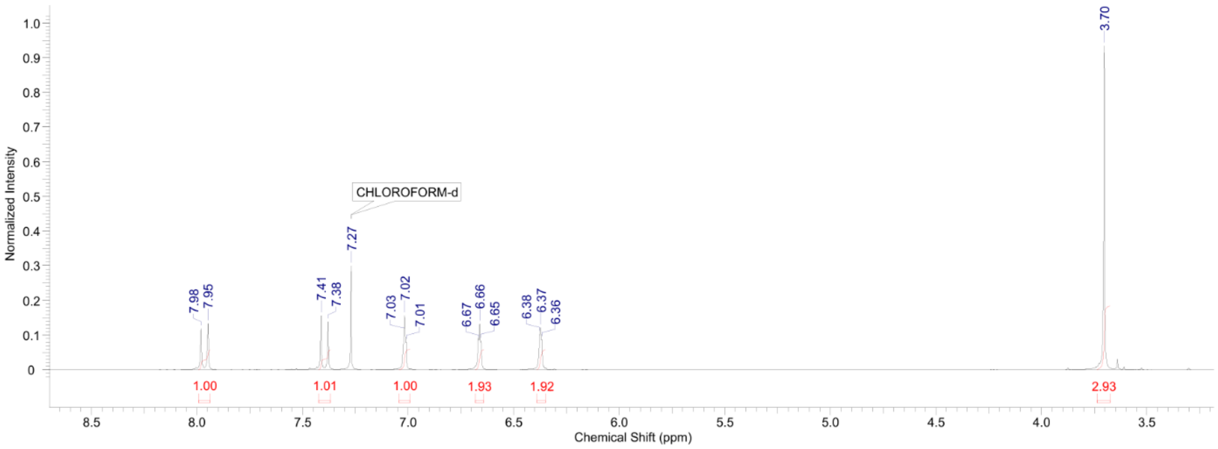

Figure S3. ^{1}H NMR spectrum of 1-methyl-3-(*trans*-2-nitrovinyl)-Δ^{3}-pyrroline (**3a**).

^{13}C NMR

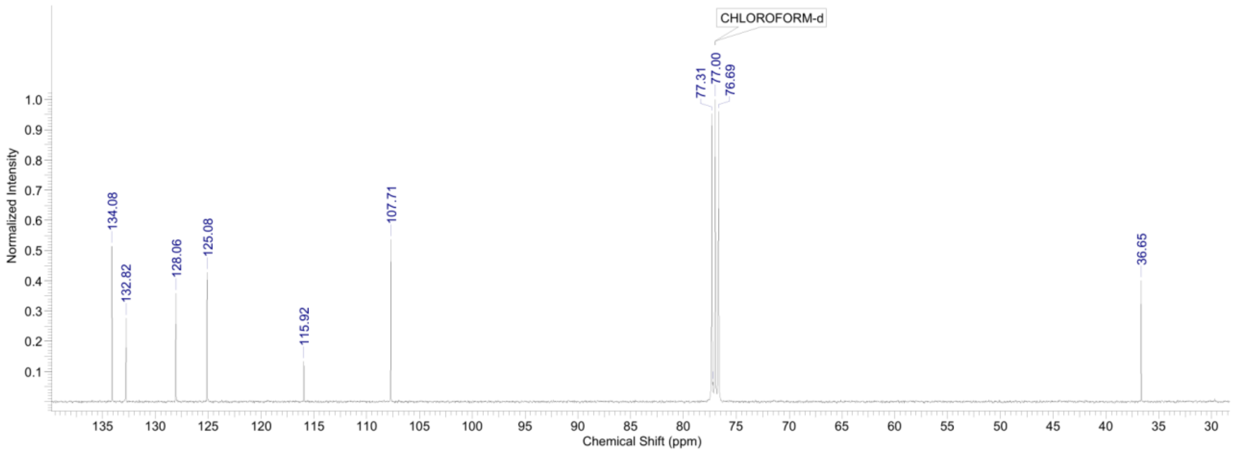

Figure S4. ^{13}C NMR spectrum of 1-methyl-3-(*trans*-2-nitrovinyl)-Δ^{3}-pyrroline (**3a**).

HMQC

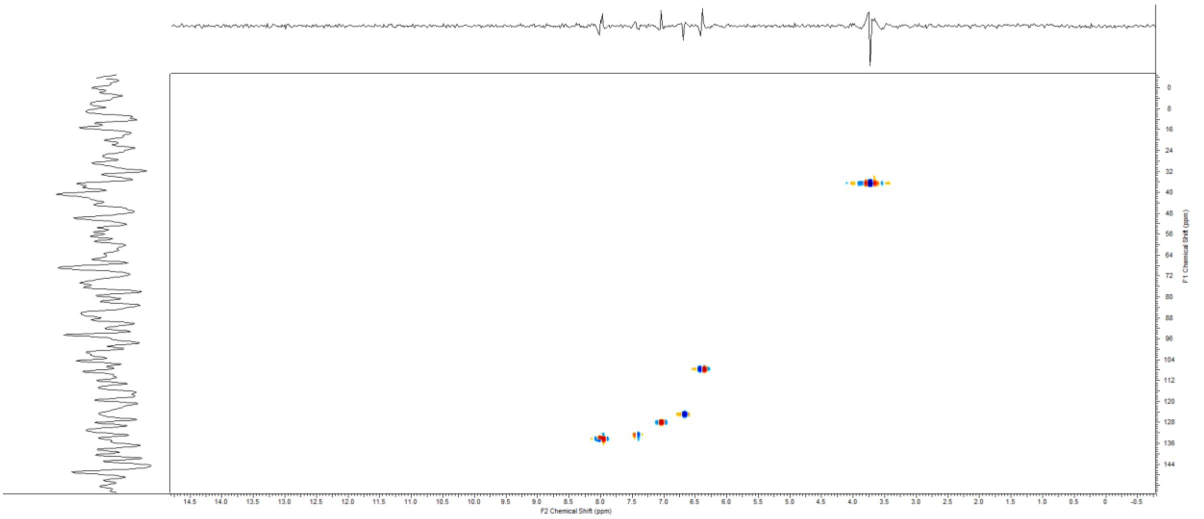

Figure S5. 2D ^{1}H-^{13}C HMQC NMR spectrum of 1-methyl-3-(*trans*-2-nitrovinyl)-Δ^{3}-pyrroline (**3a**).

Table S1. Cartesian coordinates of (1*E*,3*E*)-1,4-dinitro-1,3-butadiene (**1**) (B3LYP/6-31G(d), gas phase).

Center	Coordinates (Angstroms)		
	X	Y	Z
C	-0.67478800	0.26019800	-0.00001300
C	-1.74894700	-0.54117400	0.00007900
H	-0.83429000	1.33511200	-0.00008100
N	-3.09583400	0.01451300	0.00011100
O	-3.23204000	1.23800400	0.00005000
O	-4.01099700	-0.80958200	0.00019300
C	0.67483900	-0.26016800	-0.00001600
H	0.83434300	-1.33508500	0.00005400
C	1.74899200	0.54120400	-0.00011100
N	3.09584100	-0.01452300	-0.00012600
O	4.01101300	0.80956700	-0.00019400
O	3.23192900	-1.23803100	0.00001700
H	-1.74652700	-1.62326800	0.00015900
H	1.74660400	1.62329900	-0.00019200

Table S2. Cartesian coordinates of N-methyl azomethine ylide (**2**) (B3LYP/6-31G(d), gas phase).

Center	Coordinates (Angstroms)		
	X	Y	Z
C	-0.710539	-1.207047	-0.00295
C	-0.709039	1.207894	-0.002882
H	-1.788147	-1.268999	0.047181
H	-0.086684	-2.085684	-0.011042
H	-0.084075	2.085743	-0.011332
H	-1.786566	1.271202	0.047231
N	-0.13311	0.000066	-0.019536
C	1.346378	-0.000805	0.00999
H	1.713952	0.887717	-0.50449
H	1.68955	-0.000041	1.047141
H	1.712938	-0.890653	-0.502896
